# {μ-*N*,*N*,*N*′,*N*′-Tetra­kis[(diphenyl­phosphan­yl)meth­yl]benzene-1,4-diamine}­bis­[(2,2′-bipyrid­yl)copper(I)] bis­(tetra­fluoridoborate)

**DOI:** 10.1107/S1600536811029333

**Published:** 2011-07-23

**Authors:** Lun-Zhong Luo, Zong-Wei Yang, Zhi-Bin Wang, Zhang Hong

**Affiliations:** a SiChuan College of Chemical Technology, Luzhou 646005, People’s Republic of China

## Abstract

In the title compound, [Cu_2_(C_10_H_8_N_2_)_2_(C_58_H_52_N_2_P_4_)](BF_4_)_2_, the dinuclear cation lies on an inversion centre. The Cu^I^ atom is coordinated by two N atoms from a 2,2′-bipyridine ligand and two P atoms from an *N*,*N*,*N*′,*N*′-tetra­kis­[(diphenyl­phos­phan­yl)meth­yl]benzene-1,4-diamine ligand in a distorted tetra­hedral geometry. In the crystal, inter­molecular C—H⋯F hydrogen bonds link the ions into layers parallel to [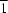
01]. π–π inter­actions [centroid–centroid distance = 3.668 (4) Å] are also observed. One F atom of the anion is disordered over two orientations with a refined occupancy ratio of 0.675 (13):0.325 (13).

## Related literature

For the synthesis, structure and applications of related copper(I) complexes, see: Chan *et al.* (1998 ▶); Chen *et al.* (2009 ▶); Linfoot *et al.* (2010 ▶); Yang *et al.* (2005 ▶); Zhang *et al.* (2007 ▶).
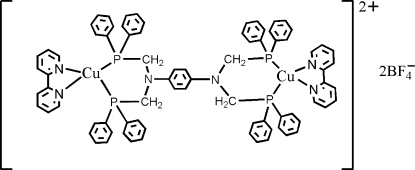


## Experimental

### 

#### Crystal data

[Cu_2_(C_10_H_8_N_2_)_2_(C_58_H_52_N_2_P_4_)](BF_4_)_2_
*M* *_r_* = 1513.96
Monoclinic, 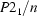

*a* = 9.912 (6) Å
*b* = 20.472 (10) Å
*c* = 17.938 (10) Å
β = 91.630 (7)°
*V* = 3638 (3) Å^3^
*Z* = 2
Mo *K*α radiation
μ = 0.74 mm^−1^
*T* = 293 K
0.20 × 0.20 × 0.20 mm

#### Data collection

Rigaky Mercury CCD diffractometer
Absorption correction: multi-scan (*ABSCOR*; Higashi, 1995 ▶) *T*_min_ = 0.866, *T*_max_ = 1.000
31355 measured reflections
6395 independent reflections
4944 reflections with *I* > 2σ(*I*)
*R*_int_ = 0.070

#### Refinement

*R*[*F* ^2^ > 2σ(*F* ^2^)] = 0.065
*wR*(*F* ^2^) = 0.173
*S* = 1.02
6395 reflections
451 parameters
11 restraints
H-atom parameters constrained
Δρ_max_ = 0.64 e Å^−3^
Δρ_min_ = −0.48 e Å^−3^

### 

Data collection: *CrystalClear-SM Expert* (Rigaku, 2009 ▶); cell refinement: *CrystalClear-SM Expert*; data reduction: *CrystalClear-SM Expert*; program(s) used to solve structure: *SHELXS97* (Sheldrick, 2008 ▶); program(s) used to refine structure: *SHELXL97* (Sheldrick, 2008 ▶); molecular graphics: *SHELXTL* (Sheldrick, 2008 ▶); software used to prepare material for publication: *SHELXTL*.

## Supplementary Material

Crystal structure: contains datablock(s) I, global. DOI: 10.1107/S1600536811029333/rz2624sup1.cif

Structure factors: contains datablock(s) I. DOI: 10.1107/S1600536811029333/rz2624Isup2.hkl

Additional supplementary materials:  crystallographic information; 3D view; checkCIF report

## Figures and Tables

**Table 1:** Hydrogen-bond geometry (Å, °)

*D*—H⋯*A*	*D*—H	H⋯*A*	*D*⋯*A*	*D*—H⋯*A*
C20—H20*A*⋯F1*B*^i^	0.93	2.43	3.36 (2)	171
C30—H30*A*⋯F2^ii^	0.93	2.31	3.216 (9)	164
C33—H33*A*⋯F3^iii^	0.93	2.42	3.319 (8)	161

Symmetry codes: (i) 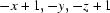; (ii) 
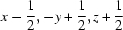
; (iii) 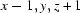.

**Fig. 1.**
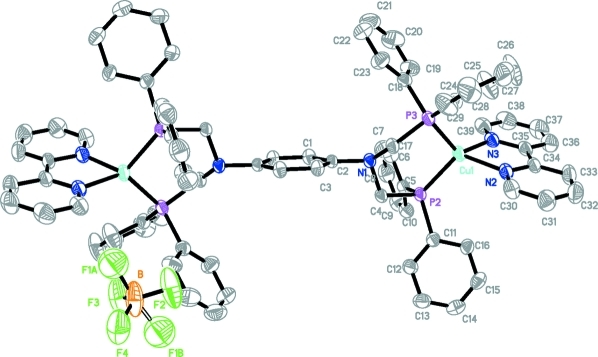
The molecular structure of title compound with displacement ellipsoids drawn at 30% probability level. Hydrogen atoms are omitted for clarity. Unlabelled atoms are related to the labelled atoms by the symmetry operation 1-x, -y, 1-z.

**Fig. 2.**
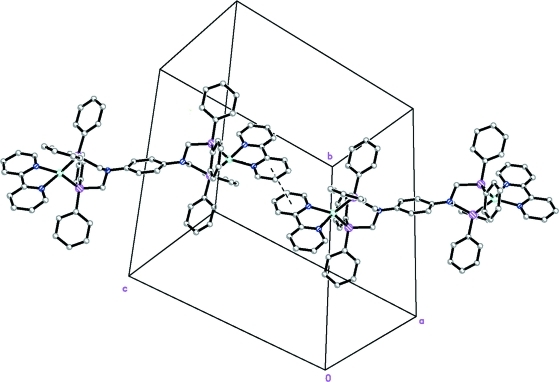
Partial packing diagram of the title compound showing a π–π interaction as dashed line. Hydrogen atoms are omitted for clarity.

## supplementary crystallographic information

### Comment

Copper(I) complexes containing phosphine and nitrogen ligands have been reported to possess catalytic and luminescent properties (Chan *et al.*, 1998; Chen *et al.*, 2009; Linfoot *et al.*, 2010; Yang *et al.*, 2005; Zhang *et al.*, 2007). As a contribution to this research field, we have synthesized the new dinuclear copper(I) title complex and report its crystal structure herein.

In the title compound (Fig. 1), the dinuclear cation has crystallographically imposed inversion symmetry, the central benzene ring of the *N*,*N*,*N*',*N*'-tetra[(diphenylphosphanyl)-methyl]benzene-1,4-diamine ligand (dpppda) lying about a centre of symmetry. Each copper(I) atom adopts a distorted tetrahedral geometry provided by two N atoms from a 2,2'-bipyridine ligand and two P atoms from the dpppda ligand. The Cu—P and Cu—N bond distances are in the range 2.2175 (17)–2.2198 (16) and 2.039 (4)–2.050 (4) Å, respectively. In the crystal structure cations and anions are linked by C—H···F hydrogen bonds (Table 1) into layers parallel to the [1 0 1] plane. π–π interactions involving the N3/C35-C39 rings of adjacent 2,2'-bipyridine ligands (centroid-to-centroid distance = 3.668 (4) Å) are also observed (Fig. 2).

### Experimental

To a solution of 2,2'-bipyridine (0.0312 g, 0.2 mmol) and *N*,*N*,*N*',*N*'-tetra[(diphenylphosphanyl)-methyl]benzene-1,4-diamine (0.0900 g, 0.10 mmol) in CH_3_CN (5 ml) Cu(CH_3_CN)_4_]BF_4_(0.0656 g, 0.2 mmol) was added with stirring. The resulting yellow solution was allowed to stir for 0.5 h. Block-shaped yellow crystals suitable for X-ray analysis were formed by slow diffusion of diethyl ether into the solution (yield: 30%).

### Refinement

All hydrogen atoms were generated geometrically and refined using a riding model, with C—H = 0.93–0.97 Å, and with *U*_iso_(H) = 1.2*U*_eq_(C). The F1 atom is disordered over two orientations, which were refined isotropically with occupancy ratio of 0.675 (13):0.325 (13). The B–F bond lengths in the anion were restrained to 1.32 (2) Å. The displacement parameters of the C25 atom were restrained to be isotropic by means of the instruction ISOR (tolerance 0.01) in *SHELXL-97*.

### Crystal data

**Table:**

[Cu_2_(C_10_H_8_N_2_)_2_(C_58_H_52_N_2_P_4_)](BF_4_)_2_	*F*(000) = 1556
*M**_r_* = 1513.96	*D*_x_ = 1.382 Mg m^−^^3^
Monoclinic, *P*2_1_/*n*	Mo *K*α radiation, λ = 0.71073 Å
Hall symbol: -P 2yn	Cell parameters from 7518 reflections
*a* = 9.912 (6) Å	θ = 2.1–27.5°
*b* = 20.472 (10) Å	µ = 0.74 mm^−^^1^
*c* = 17.938 (10) Å	*T* = 293 K
β = 91.630 (7)°	Prism, yellow
*V* = 3638 (3) Å^3^	0.20 × 0.20 × 0.20 mm
*Z* = 2	

### Data collection

**Table:**

Rigaky Mercury CCD diffractometer	6395 independent reflections
Radiation source: fine-focus sealed tube	4944 reflections with *I* > 2σ(*I*)
graphite	*R*_int_ = 0.070
Detector resolution: 13.6612 pixels mm^-1^	θ_max_ = 25.0°, θ_min_ = 2.5°
ω scans	*h* = −11→11
Absorption correction: multi-scan (*ABSCOR*; Higashi, 1995)	*k* = −24→24
*T*_min_ = 0.866, *T*_max_ = 1.000	*l* = −21→21
31355 measured reflections	

### Refinement

**Table:**

Refinement on *F*^2^	Primary atom site location: structure-invariant direct methods
Least-squares matrix: full	Secondary atom site location: difference Fourier map
*R*[*F*^2^ > 2σ(*F*^2^)] = 0.065	Hydrogen site location: inferred from neighbouring sites
*wR*(*F*^2^) = 0.173	H-atom parameters constrained
*S* = 1.02	*w* = 1/[σ^2^(*F*_o_^2^) + (0.079*P*)^2^ + 4.5666*P*] where *P* = (*F*_o_^2^ + 2*F*_c_^2^)/3
6395 reflections	(Δ/σ)_max_ < 0.001
451 parameters	Δρ_max_ = 0.64 e Å^−^^3^
11 restraints	Δρ_min_ = −0.48 e Å^−^^3^

### Special details

**Table:**

Geometry. All e.s.d.'s (except the e.s.d. in the dihedral angle between two l.s. planes) are estimated using the full covariance matrix. The cell e.s.d.'s are taken into account individually in the estimation of e.s.d.'s in distances, angles and torsion angles; correlations between e.s.d.'s in cell parameters are only used when they are defined by crystal symmetry. An approximate (isotropic) treatment of cell e.s.d.'s is used for estimating e.s.d.'s involving l.s. planes.
Refinement. Refinement of *F*^2^ against ALL reflections. The weighted *R*-factor *wR* and goodness of fit *S* are based on *F*^2^, conventional *R*-factors *R* are based on *F*, with *F* set to zero for negative *F*^2^. The threshold expression of *F*^2^ > σ(*F*^2^) is used only for calculating *R*-factors(gt) *etc*. and is not relevant to the choice of reflections for refinement. *R*-factors based on *F*^2^ are statistically about twice as large as those based on *F*, and *R*- factors based on ALL data will be even larger.

### Fractional atomic coordinates and isotropic or equivalent isotropic displacement parameters (Å^2^)

**Table:**

	*x*	*y*	*z*	*U*_iso_*/*U*_eq_	Occ. (<1)
Cu1	0.14600 (5)	0.06738 (3)	0.78116 (3)	0.0534 (2)	
P2	0.07845 (11)	0.06121 (6)	0.66233 (6)	0.0487 (3)	
P3	0.36642 (11)	0.05056 (6)	0.77515 (6)	0.0488 (3)	
N1	0.3460 (3)	0.03687 (17)	0.62451 (18)	0.0463 (8)	
N3	0.0160 (4)	0.0323 (2)	0.8576 (2)	0.0574 (9)	
C4	0.2202 (4)	0.0676 (2)	0.5974 (2)	0.0523 (11)	
H4A	0.1930	0.0476	0.5504	0.063*	
H4B	0.2376	0.1134	0.5880	0.063*	
C17	0.4184 (4)	0.0759 (2)	0.6819 (2)	0.0492 (10)	
H17A	0.3985	0.1219	0.6745	0.059*	
H17B	0.5149	0.0698	0.6776	0.059*	
C2	0.4275 (4)	0.0188 (2)	0.5622 (2)	0.0420 (9)	
C11	−0.0408 (4)	0.1208 (2)	0.6247 (2)	0.0514 (10)	
N2	0.1007 (4)	0.1529 (2)	0.8344 (2)	0.0584 (9)	
C35	−0.0349 (5)	0.0794 (3)	0.9017 (2)	0.0616 (12)	
C3	0.5274 (4)	0.0590 (2)	0.5345 (2)	0.0485 (10)	
H3A	0.5462	0.0989	0.5572	0.058*	
C5	−0.0030 (4)	−0.0164 (2)	0.6417 (2)	0.0538 (10)	
C16	−0.1308 (4)	0.1484 (2)	0.6721 (3)	0.0587 (11)	
H16A	−0.1273	0.1372	0.7224	0.070*	
C34	0.0164 (4)	0.1461 (2)	0.8906 (2)	0.0575 (11)	
C18	0.4462 (4)	−0.0284 (2)	0.7868 (2)	0.0543 (11)	
C10	−0.1359 (5)	−0.0203 (3)	0.6130 (3)	0.0625 (12)	
H10A	−0.1808	0.0177	0.5985	0.075*	
C36	−0.1313 (6)	0.0644 (3)	0.9537 (3)	0.0850 (17)	
H36A	−0.1653	0.0967	0.9844	0.102*	
C12	−0.0462 (5)	0.1384 (3)	0.5504 (3)	0.0702 (14)	
H12A	0.0133	0.1199	0.5173	0.084*	
C15	−0.2269 (5)	0.1926 (3)	0.6460 (3)	0.0698 (14)	
H15A	−0.2887	0.2102	0.6785	0.084*	
C33	−0.0191 (6)	0.1995 (3)	0.9335 (3)	0.0823 (16)	
H33A	−0.0750	0.1938	0.9738	0.099*	
C13	−0.1408 (5)	0.1841 (3)	0.5252 (3)	0.0777 (15)	
H13A	−0.1428	0.1969	0.4754	0.093*	
C9	−0.2007 (6)	−0.0791 (3)	0.6060 (3)	0.0799 (16)	
H9A	−0.2887	−0.0805	0.5867	0.096*	
C6	0.0604 (6)	−0.0745 (3)	0.6634 (3)	0.0733 (14)	
H6A	0.1481	−0.0735	0.6833	0.088*	
C19	0.3733 (6)	−0.0810 (3)	0.8127 (3)	0.0756 (15)	
H19A	0.2828	−0.0755	0.8237	0.091*	
C8	−0.1382 (7)	−0.1347 (3)	0.6268 (3)	0.0890 (18)	
H8A	−0.1838	−0.1743	0.6218	0.107*	
C21	0.5626 (9)	−0.1505 (3)	0.8062 (4)	0.102 (2)	
H21A	0.6011	−0.1916	0.8121	0.123*	
C30	0.1472 (6)	0.2130 (3)	0.8191 (3)	0.0769 (15)	
H30A	0.2062	0.2181	0.7802	0.092*	
C23	0.5806 (6)	−0.0389 (3)	0.7710 (3)	0.0792 (15)	
H23A	0.6321	−0.0045	0.7535	0.095*	
C38	−0.1238 (7)	−0.0460 (4)	0.9148 (4)	0.095 (2)	
H38A	−0.1529	−0.0891	0.9186	0.114*	
C14	−0.2306 (5)	0.2101 (3)	0.5729 (3)	0.0717 (14)	
H14A	−0.2947	0.2400	0.5555	0.086*	
C22	0.6391 (7)	−0.0995 (4)	0.7807 (4)	0.096 (2)	
H22A	0.7295	−0.1058	0.7702	0.115*	
C7	−0.0071 (8)	−0.1341 (3)	0.6556 (4)	0.0917 (19)	
H7A	0.0354	−0.1729	0.6696	0.110*	
C39	−0.0272 (5)	−0.0287 (3)	0.8640 (3)	0.0748 (14)	
H39A	0.0084	−0.0608	0.8336	0.090*	
C37	−0.1751 (7)	0.0016 (4)	0.9590 (4)	0.102 (2)	
H37A	−0.2407	−0.0089	0.9931	0.123*	
C31	0.1106 (7)	0.2672 (3)	0.8592 (4)	0.0900 (18)	
H31A	0.1427	0.3083	0.8466	0.108*	
C32	0.0276 (7)	0.2599 (3)	0.9169 (4)	0.0947 (19)	
H32A	0.0027	0.2959	0.9450	0.114*	
F3	0.8501 (6)	0.1767 (2)	0.0998 (3)	0.154 (2)	
C1	0.4009 (4)	−0.0397 (2)	0.5269 (2)	0.0496 (10)	
H1A	0.3337	−0.0668	0.5447	0.059*	
C20	0.4326 (9)	−0.1410 (3)	0.8225 (4)	0.104 (2)	
H20A	0.3823	−0.1756	0.8406	0.125*	
B	0.9019 (11)	0.2287 (5)	0.1340 (5)	0.126 (4)	
F4	0.9421 (6)	0.2774 (2)	0.0915 (3)	0.162 (2)	
F1A	1.0328 (10)	0.1992 (4)	0.1493 (5)	0.168 (5)*	0.675 (13)
F1B	0.7738 (19)	0.2625 (10)	0.1282 (13)	0.189 (11)*	0.325 (13)
C24	0.4638 (4)	0.1026 (2)	0.8380 (2)	0.0582 (11)	
C29	0.5659 (7)	0.1438 (3)	0.8177 (4)	0.098 (2)	
H29A	0.5879	0.1454	0.7677	0.118*	
C28	0.6372 (9)	0.1826 (4)	0.8672 (5)	0.121 (3)	
H28A	0.7097	0.2077	0.8520	0.145*	
C25	0.4326 (8)	0.1042 (5)	0.9100 (4)	0.127 (3)	
H25A	0.3619	0.0784	0.9262	0.152*	
C27	0.5981 (9)	0.1832 (4)	0.9393 (5)	0.116 (3)	
H27A	0.6395	0.2120	0.9729	0.139*	
C26	0.5039 (9)	0.1441 (6)	0.9625 (4)	0.146 (4)	
H26A	0.4837	0.1425	1.0128	0.176*	
F2	0.8636 (8)	0.2407 (4)	0.1998 (3)	0.209 (3)	

### Atomic displacement parameters (Å^2^)

**Table:**

	*U*^11^	*U*^22^	*U*^33^	*U*^12^	*U*^13^	*U*^23^
Cu1	0.0458 (3)	0.0768 (4)	0.0380 (3)	0.0015 (2)	0.0080 (2)	−0.0052 (2)
P2	0.0386 (5)	0.0697 (8)	0.0379 (6)	0.0084 (5)	0.0029 (4)	−0.0040 (5)
P3	0.0427 (6)	0.0680 (7)	0.0356 (5)	0.0001 (5)	0.0021 (4)	−0.0035 (5)
N1	0.0336 (16)	0.066 (2)	0.0394 (17)	0.0025 (15)	0.0044 (14)	−0.0049 (15)
N3	0.049 (2)	0.079 (3)	0.044 (2)	−0.0034 (19)	0.0035 (17)	0.0022 (18)
C4	0.039 (2)	0.082 (3)	0.036 (2)	0.011 (2)	0.0034 (17)	−0.0010 (19)
C17	0.044 (2)	0.064 (3)	0.040 (2)	−0.0028 (18)	0.0056 (18)	−0.0035 (18)
C2	0.0346 (19)	0.057 (2)	0.0347 (19)	0.0059 (17)	0.0049 (16)	0.0001 (17)
C11	0.040 (2)	0.063 (3)	0.051 (2)	0.0016 (18)	0.0017 (18)	−0.002 (2)
N2	0.053 (2)	0.078 (3)	0.044 (2)	0.0000 (19)	0.0047 (17)	−0.0053 (18)
C35	0.047 (2)	0.096 (4)	0.041 (2)	0.007 (2)	0.006 (2)	0.005 (2)
C3	0.048 (2)	0.052 (2)	0.046 (2)	−0.0047 (18)	0.0075 (18)	−0.0043 (18)
C5	0.053 (2)	0.068 (3)	0.040 (2)	0.008 (2)	0.0021 (19)	−0.0027 (19)
C16	0.046 (2)	0.074 (3)	0.056 (3)	0.008 (2)	0.004 (2)	−0.009 (2)
C34	0.052 (3)	0.083 (3)	0.037 (2)	0.007 (2)	0.0037 (19)	−0.006 (2)
C18	0.051 (2)	0.066 (3)	0.045 (2)	0.000 (2)	−0.006 (2)	−0.002 (2)
C10	0.054 (3)	0.077 (3)	0.056 (3)	−0.006 (2)	0.002 (2)	0.001 (2)
C36	0.065 (3)	0.121 (5)	0.070 (4)	0.003 (3)	0.027 (3)	0.016 (3)
C12	0.058 (3)	0.096 (4)	0.057 (3)	0.019 (3)	0.011 (2)	0.012 (3)
C15	0.050 (3)	0.072 (3)	0.087 (4)	0.012 (2)	0.005 (3)	−0.011 (3)
C33	0.079 (4)	0.107 (5)	0.062 (3)	0.009 (3)	0.016 (3)	−0.019 (3)
C13	0.068 (3)	0.092 (4)	0.073 (3)	0.016 (3)	0.002 (3)	0.024 (3)
C9	0.072 (4)	0.096 (4)	0.073 (4)	−0.013 (3)	0.007 (3)	−0.001 (3)
C6	0.076 (3)	0.078 (4)	0.066 (3)	0.017 (3)	−0.001 (3)	0.000 (3)
C19	0.072 (3)	0.077 (4)	0.077 (4)	−0.009 (3)	−0.005 (3)	0.018 (3)
C8	0.102 (5)	0.085 (4)	0.080 (4)	−0.016 (4)	0.015 (4)	−0.002 (3)
C21	0.136 (7)	0.070 (4)	0.099 (5)	0.024 (4)	−0.022 (5)	0.005 (3)
C30	0.086 (4)	0.083 (4)	0.063 (3)	−0.012 (3)	0.013 (3)	−0.006 (3)
C23	0.065 (3)	0.079 (4)	0.094 (4)	0.011 (3)	0.010 (3)	0.010 (3)
C38	0.085 (4)	0.112 (5)	0.088 (4)	−0.025 (4)	0.007 (4)	0.027 (4)
C14	0.057 (3)	0.063 (3)	0.095 (4)	0.008 (2)	−0.004 (3)	0.010 (3)
C22	0.089 (4)	0.102 (5)	0.097 (5)	0.039 (4)	0.000 (4)	0.003 (4)
C7	0.129 (6)	0.059 (4)	0.088 (4)	0.020 (3)	0.012 (4)	0.003 (3)
C39	0.072 (3)	0.090 (4)	0.062 (3)	−0.010 (3)	0.003 (3)	0.007 (3)
C37	0.079 (4)	0.141 (7)	0.089 (5)	−0.012 (4)	0.035 (4)	0.032 (4)
C31	0.110 (5)	0.073 (4)	0.087 (4)	−0.010 (3)	0.002 (4)	−0.014 (3)
C32	0.106 (5)	0.093 (5)	0.086 (4)	0.010 (4)	0.006 (4)	−0.032 (4)
F3	0.194 (5)	0.150 (4)	0.123 (3)	−0.071 (4)	0.067 (3)	−0.056 (3)
C1	0.043 (2)	0.056 (3)	0.050 (2)	−0.0088 (18)	0.0067 (19)	−0.0047 (19)
C20	0.123 (6)	0.073 (4)	0.116 (6)	−0.011 (4)	−0.008 (5)	0.027 (4)
B	0.130 (8)	0.122 (7)	0.129 (8)	−0.063 (6)	0.080 (7)	−0.049 (6)
F4	0.201 (5)	0.119 (4)	0.171 (5)	−0.015 (4)	0.083 (4)	−0.025 (3)
C24	0.048 (2)	0.079 (3)	0.047 (2)	0.004 (2)	−0.005 (2)	−0.014 (2)
C29	0.123 (5)	0.095 (5)	0.076 (4)	−0.038 (4)	−0.021 (4)	−0.005 (3)
C28	0.144 (7)	0.099 (5)	0.117 (6)	−0.029 (5)	−0.023 (5)	−0.029 (5)
C25	0.123 (5)	0.187 (7)	0.070 (4)	−0.058 (5)	0.008 (4)	−0.028 (4)
C27	0.121 (6)	0.103 (6)	0.123 (7)	0.011 (5)	−0.028 (5)	−0.057 (5)
C26	0.138 (7)	0.236 (11)	0.066 (4)	−0.041 (7)	0.012 (5)	−0.062 (6)
F2	0.266 (8)	0.264 (8)	0.101 (4)	−0.030 (6)	0.057 (5)	−0.084 (4)

### Geometric parameters (Å, °)

**Table:**

Cu1—N3	2.039 (4)	C13—H13A	0.9300
Cu1—N2	2.050 (4)	C9—C8	1.345 (8)
Cu1—P3	2.2175 (17)	C9—H9A	0.9300
Cu1—P2	2.2198 (16)	C6—C7	1.397 (8)
P2—C11	1.816 (4)	C6—H6A	0.9300
P2—C5	1.816 (5)	C19—C20	1.372 (8)
P2—C4	1.855 (4)	C19—H19A	0.9300
P3—C18	1.810 (5)	C8—C7	1.385 (9)
P3—C24	1.810 (5)	C8—H8A	0.9300
P3—C17	1.839 (4)	C21—C20	1.344 (10)
N1—C2	1.446 (5)	C21—C22	1.375 (10)
N1—C4	1.467 (5)	C21—H21A	0.9300
N1—C17	1.473 (5)	C30—C31	1.376 (8)
N3—C39	1.327 (7)	C30—H30A	0.9300
N3—C35	1.353 (6)	C23—C22	1.378 (8)
C4—H4A	0.9700	C23—H23A	0.9300
C4—H4B	0.9700	C38—C37	1.365 (9)
C17—H17A	0.9700	C38—C39	1.386 (8)
C17—H17B	0.9700	C38—H38A	0.9300
C2—C1	1.377 (6)	C14—H14A	0.9300
C2—C3	1.390 (5)	C22—H22A	0.9300
C11—C16	1.371 (6)	C7—H7A	0.9300
C11—C12	1.381 (6)	C39—H39A	0.9300
N2—C34	1.335 (5)	C37—H37A	0.9300
N2—C30	1.344 (7)	C31—C32	1.350 (9)
C35—C36	1.389 (7)	C31—H31A	0.9300
C35—C34	1.474 (7)	C32—H32A	0.9300
C3—C1^i^	1.385 (6)	F3—B	1.325 (8)
C3—H3A	0.9300	C1—C3^i^	1.385 (6)
C5—C6	1.395 (7)	C1—H1A	0.9300
C5—C10	1.403 (6)	C20—H20A	0.9300
C16—C15	1.386 (6)	B—F2	1.275 (8)
C16—H16A	0.9300	B—F4	1.323 (9)
C34—C33	1.389 (7)	B—F1B	1.447 (15)
C18—C19	1.384 (7)	B—F1A	1.451 (12)
C18—C23	1.387 (7)	F1B—F2	1.61 (2)
C10—C9	1.367 (7)	C24—C25	1.337 (8)
C10—H10A	0.9300	C24—C29	1.374 (8)
C36—C37	1.360 (9)	C29—C28	1.373 (8)
C36—H36A	0.9300	C29—H29A	0.9300
C12—C13	1.390 (7)	C28—C27	1.361 (11)
C12—H12A	0.9300	C28—H28A	0.9300
C15—C14	1.359 (7)	C25—C26	1.420 (10)
C15—H15A	0.9300	C25—H25A	0.9300
C33—C32	1.356 (9)	C27—C26	1.307 (11)
C33—H33A	0.9300	C27—H27A	0.9300
C13—C14	1.361 (7)	C26—H26A	0.9300
			
N3—Cu1—N2	80.60 (16)	C8—C9—C10	120.6 (6)
N3—Cu1—P3	128.36 (11)	C8—C9—H9A	119.7
N2—Cu1—P3	112.60 (11)	C10—C9—H9A	119.7
N3—Cu1—P2	116.53 (11)	C5—C6—C7	120.3 (6)
N2—Cu1—P2	115.57 (11)	C5—C6—H6A	119.8
P3—Cu1—P2	102.42 (5)	C7—C6—H6A	119.8
C11—P2—C5	103.4 (2)	C20—C19—C18	120.9 (6)
C11—P2—C4	102.5 (2)	C20—C19—H19A	119.6
C5—P2—C4	105.9 (2)	C18—C19—H19A	119.6
C11—P2—Cu1	119.61 (15)	C9—C8—C7	121.0 (6)
C5—P2—Cu1	111.45 (14)	C9—C8—H8A	119.5
C4—P2—Cu1	112.67 (14)	C7—C8—H8A	119.5
C18—P3—C24	103.3 (2)	C20—C21—C22	120.3 (6)
C18—P3—C17	103.0 (2)	C20—C21—H21A	119.9
C24—P3—C17	104.0 (2)	C22—C21—H21A	119.9
C18—P3—Cu1	124.13 (16)	N2—C30—C31	122.3 (5)
C24—P3—Cu1	112.77 (15)	N2—C30—H30A	118.8
C17—P3—Cu1	107.60 (14)	C31—C30—H30A	118.8
C2—N1—C4	110.0 (3)	C22—C23—C18	121.1 (6)
C2—N1—C17	114.0 (3)	C22—C23—H23A	119.5
C4—N1—C17	113.0 (3)	C18—C23—H23A	119.5
C39—N3—C35	119.5 (4)	C37—C38—C39	118.3 (6)
C39—N3—Cu1	127.0 (4)	C37—C38—H38A	120.8
C35—N3—Cu1	113.3 (3)	C39—C38—H38A	120.8
N1—C4—P2	114.5 (3)	C15—C14—C13	120.3 (5)
N1—C4—H4A	108.6	C15—C14—H14A	119.9
P2—C4—H4A	108.6	C13—C14—H14A	119.9
N1—C4—H4B	108.6	C21—C22—C23	119.4 (6)
P2—C4—H4B	108.6	C21—C22—H22A	120.3
H4A—C4—H4B	107.6	C23—C22—H22A	120.3
N1—C17—P3	109.8 (3)	C8—C7—C6	119.2 (6)
N1—C17—H17A	109.7	C8—C7—H7A	120.4
P3—C17—H17A	109.7	C6—C7—H7A	120.4
N1—C17—H17B	109.7	N3—C39—C38	121.9 (6)
P3—C17—H17B	109.7	N3—C39—H39A	119.1
H17A—C17—H17B	108.2	C38—C39—H39A	119.1
C1—C2—C3	118.7 (4)	C36—C37—C38	120.7 (6)
C1—C2—N1	118.3 (3)	C36—C37—H37A	119.7
C3—C2—N1	123.0 (4)	C38—C37—H37A	119.7
C16—C11—C12	118.9 (4)	C32—C31—C30	119.2 (6)
C16—C11—P2	118.3 (3)	C32—C31—H31A	120.4
C12—C11—P2	122.8 (3)	C30—C31—H31A	120.4
C34—N2—C30	118.3 (4)	C31—C32—C33	119.2 (6)
C34—N2—Cu1	114.3 (3)	C31—C32—H32A	120.4
C30—N2—Cu1	127.3 (3)	C33—C32—H32A	120.4
N3—C35—C36	120.7 (5)	C2—C1—C3^i^	121.1 (4)
N3—C35—C34	116.4 (4)	C2—C1—H1A	119.4
C36—C35—C34	122.9 (5)	C3^i^—C1—H1A	119.4
C1^i^—C3—C2	120.2 (4)	C21—C20—C19	120.7 (6)
C1^i^—C3—H3A	119.9	C21—C20—H20A	119.6
C2—C3—H3A	119.9	C19—C20—H20A	119.6
C6—C5—C10	117.7 (5)	F2—B—F4	119.4 (8)
C6—C5—P2	119.6 (4)	F2—B—F3	117.5 (7)
C10—C5—P2	122.2 (4)	F4—B—F3	117.2 (8)
C11—C16—C15	120.9 (5)	F2—B—F1B	72.0 (10)
C11—C16—H16A	119.6	F4—B—F1B	82.9 (12)
C15—C16—H16A	119.6	F3—B—F1B	91.4 (11)
N2—C34—C33	120.7 (5)	F2—B—F1A	101.1 (10)
N2—C34—C35	115.2 (4)	F4—B—F1A	98.0 (7)
C33—C34—C35	124.1 (5)	F3—B—F1A	95.0 (8)
C19—C18—C23	117.6 (5)	F1B—B—F1A	172.3 (12)
C19—C18—P3	120.2 (4)	B—F1B—F2	49.0 (7)
C23—C18—P3	122.2 (4)	C25—C24—C29	115.7 (5)
C9—C10—C5	121.2 (5)	C25—C24—P3	118.8 (5)
C9—C10—H10A	119.4	C29—C24—P3	125.5 (4)
C5—C10—H10A	119.4	C28—C29—C24	123.6 (7)
C37—C36—C35	118.8 (6)	C28—C29—H29A	118.2
C37—C36—H36A	120.6	C24—C29—H29A	118.2
C35—C36—H36A	120.6	C27—C28—C29	117.6 (8)
C11—C12—C13	119.8 (5)	C27—C28—H28A	121.2
C11—C12—H12A	120.1	C29—C28—H28A	121.2
C13—C12—H12A	120.1	C24—C25—C26	122.1 (7)
C14—C15—C16	119.8 (5)	C24—C25—H25A	119.0
C14—C15—H15A	120.1	C26—C25—H25A	119.0
C16—C15—H15A	120.1	C26—C27—C28	121.6 (7)
C32—C33—C34	120.2 (5)	C26—C27—H27A	119.2
C32—C33—H33A	119.9	C28—C27—H27A	119.2
C34—C33—H33A	119.9	C27—C26—C25	119.1 (7)
C14—C13—C12	120.4 (5)	C27—C26—H26A	120.4
C14—C13—H13A	119.8	C25—C26—H26A	120.4
C12—C13—H13A	119.8	B—F2—F1B	59.0 (7)
			
N3—Cu1—P2—C11	78.8 (2)	N3—C35—C34—N2	4.4 (6)
N2—Cu1—P2—C11	−13.3 (2)	C36—C35—C34—N2	−174.9 (5)
P3—Cu1—P2—C11	−136.12 (17)	N3—C35—C34—C33	−176.4 (5)
N3—Cu1—P2—C5	−41.8 (2)	C36—C35—C34—C33	4.3 (8)
N2—Cu1—P2—C5	−133.93 (19)	C24—P3—C18—C19	119.0 (4)
P3—Cu1—P2—C5	103.28 (16)	C17—P3—C18—C19	−133.0 (4)
N3—Cu1—P2—C4	−160.8 (2)	Cu1—P3—C18—C19	−10.9 (5)
N2—Cu1—P2—C4	107.1 (2)	C24—P3—C18—C23	−60.7 (5)
P3—Cu1—P2—C4	−15.65 (17)	C17—P3—C18—C23	47.3 (5)
N3—Cu1—P3—C18	45.1 (2)	Cu1—P3—C18—C23	169.4 (4)
N2—Cu1—P3—C18	141.1 (2)	C6—C5—C10—C9	−0.5 (7)
P2—Cu1—P3—C18	−94.16 (17)	P2—C5—C10—C9	−172.3 (4)
N3—Cu1—P3—C24	−80.8 (2)	N3—C35—C36—C37	−0.8 (8)
N2—Cu1—P3—C24	15.2 (2)	C34—C35—C36—C37	178.5 (5)
P2—Cu1—P3—C24	139.95 (18)	C16—C11—C12—C13	0.8 (8)
N3—Cu1—P3—C17	165.1 (2)	P2—C11—C12—C13	179.5 (4)
N2—Cu1—P3—C17	−98.96 (19)	C11—C16—C15—C14	−1.4 (8)
P2—Cu1—P3—C17	25.82 (16)	N2—C34—C33—C32	2.6 (8)
N2—Cu1—N3—C39	176.7 (4)	C35—C34—C33—C32	−176.6 (5)
P3—Cu1—N3—C39	−71.9 (4)	C11—C12—C13—C14	−1.8 (9)
P2—Cu1—N3—C39	62.7 (4)	C5—C10—C9—C8	0.2 (8)
N2—Cu1—N3—C35	1.2 (3)	C10—C5—C6—C7	0.8 (7)
P3—Cu1—N3—C35	112.6 (3)	P2—C5—C6—C7	172.8 (4)
P2—Cu1—N3—C35	−112.8 (3)	C23—C18—C19—C20	0.3 (8)
C2—N1—C4—P2	155.1 (3)	P3—C18—C19—C20	−179.4 (5)
C17—N1—C4—P2	−76.2 (4)	C10—C9—C8—C7	−0.1 (9)
C11—P2—C4—N1	165.4 (3)	C34—N2—C30—C31	−0.2 (8)
C5—P2—C4—N1	−86.6 (3)	Cu1—N2—C30—C31	−179.4 (4)
Cu1—P2—C4—N1	35.5 (4)	C19—C18—C23—C22	0.0 (8)
C2—N1—C17—P3	−141.3 (3)	P3—C18—C23—C22	179.7 (5)
C4—N1—C17—P3	92.1 (4)	C16—C15—C14—C13	0.4 (8)
C18—P3—C17—N1	72.4 (3)	C12—C13—C14—C15	1.2 (9)
C24—P3—C17—N1	179.9 (3)	C20—C21—C22—C23	−1.0 (11)
Cu1—P3—C17—N1	−60.2 (3)	C18—C23—C22—C21	0.4 (10)
C4—N1—C2—C1	−83.7 (5)	C9—C8—C7—C6	0.4 (9)
C17—N1—C2—C1	148.1 (4)	C5—C6—C7—C8	−0.7 (9)
C4—N1—C2—C3	93.6 (4)	C35—N3—C39—C38	0.2 (8)
C17—N1—C2—C3	−34.6 (5)	Cu1—N3—C39—C38	−175.1 (4)
C5—P2—C11—C16	95.6 (4)	C37—C38—C39—N3	0.1 (9)
C4—P2—C11—C16	−154.5 (4)	C35—C36—C37—C38	1.0 (10)
Cu1—P2—C11—C16	−29.0 (4)	C39—C38—C37—C36	−0.7 (10)
C5—P2—C11—C12	−83.1 (4)	N2—C30—C31—C32	1.5 (10)
C4—P2—C11—C12	26.8 (5)	C30—C31—C32—C33	−0.8 (10)
Cu1—P2—C11—C12	152.3 (4)	C34—C33—C32—C31	−1.2 (10)
N3—Cu1—N2—C34	1.2 (3)	C3—C2—C1—C3^i^	0.7 (7)
P3—Cu1—N2—C34	−126.6 (3)	N1—C2—C1—C3^i^	178.0 (4)
P2—Cu1—N2—C34	116.2 (3)	C22—C21—C20—C19	1.3 (12)
N3—Cu1—N2—C30	−179.5 (4)	C18—C19—C20—C21	−0.9 (11)
P3—Cu1—N2—C30	52.7 (4)	F4—B—F1B—F2	−124.1 (7)
P2—Cu1—N2—C30	−64.5 (4)	F3—B—F1B—F2	118.6 (7)
C39—N3—C35—C36	0.2 (7)	C18—P3—C24—C25	−84.1 (6)
Cu1—N3—C35—C36	176.1 (4)	C17—P3—C24—C25	168.7 (6)
C39—N3—C35—C34	−179.1 (4)	Cu1—P3—C24—C25	52.4 (6)
Cu1—N3—C35—C34	−3.3 (5)	C18—P3—C24—C29	98.9 (5)
C1—C2—C3—C1^i^	−0.7 (7)	C17—P3—C24—C29	−8.4 (6)
N1—C2—C3—C1^i^	−177.9 (4)	Cu1—P3—C24—C29	−124.7 (5)
C11—P2—C5—C6	−179.6 (4)	C25—C24—C29—C28	2.7 (11)
C4—P2—C5—C6	73.0 (4)	P3—C24—C29—C28	179.8 (6)
Cu1—P2—C5—C6	−49.8 (4)	C24—C29—C28—C27	−4.4 (12)
C11—P2—C5—C10	−7.9 (4)	C29—C24—C25—C26	−2.3 (12)
C4—P2—C5—C10	−115.3 (4)	P3—C24—C25—C26	−179.6 (8)
Cu1—P2—C5—C10	121.8 (3)	C29—C28—C27—C26	5.7 (14)
C12—C11—C16—C15	0.8 (7)	C28—C27—C26—C25	−5.5 (16)
P2—C11—C16—C15	−178.0 (4)	C24—C25—C26—C27	3.8 (16)
C30—N2—C34—C33	−1.9 (7)	F4—B—F2—F1B	70.6 (13)
Cu1—N2—C34—C33	177.5 (4)	F3—B—F2—F1B	−81.9 (13)
C30—N2—C34—C35	177.4 (4)	F1A—B—F2—F1B	176.5 (12)
Cu1—N2—C34—C35	−3.3 (5)		

Symmetry codes: (i) −*x*+1, −*y*, −*z*+1.

### Hydrogen-bond geometry (Å, °)

**Table:**

*D*—H···*A*	*D*—H	H···*A*	*D*···*A*	*D*—H···*A*
C20—H20A···F1B^i^	0.93	2.43	3.36 (2)	171
C30—H30A···F2^ii^	0.93	2.31	3.216 (9)	164
C33—H33A···F3^iii^	0.93	2.42	3.319 (8)	161

Symmetry codes: (i) −*x*+1, −*y*, −*z*+1; (ii) *x*−1/2, −*y*+1/2, *z*+1/2; (iii) *x*−1, *y*, *z*+1.
